# Growth Hormone Overexpression Disrupts Reproductive Status Through Actions on Leptin

**DOI:** 10.3389/fendo.2018.00131

**Published:** 2018-03-27

**Authors:** Ji Chen, Mengxi Cao, Aidi Zhang, Mijuan Shi, Binbin Tao, Yongming Li, Yaping Wang, Zuoyan Zhu, Vance L. Trudeau, Wei Hu

**Affiliations:** ^1^State Key Laboratory of Freshwater Ecology and Biotechnology, Institute of Hydrobiology, Chinese Academy of Sciences, Wuhan, China; ^2^Institute of Environment and Health, Jianghan University, Wuhan, China; ^3^Department of Biology, University of Ottawa, Ottawa, ON, Canada

**Keywords:** common carp, growth hormone, reproductive regulation, energy balance, leptin

## Abstract

Growth and reproduction are closely related. Growth hormone (GH)-transgenic common carp exhibit accelerated growth and delayed reproductive development, which provides an amenable model to study hormone cross talk between the growth and reproductive axes. We analyzed the energy status and reproductive development in GH-transgenic common carp by using multi-tissue RNA sequencing, real-time-PCR, Western blotting, ELISA, immunofluorescence, and *in vitro* incubation. The expression of *gys* (glycogen synthase) and *igfbp1* (insulin-like growth factor binding protein) as well as blood glucose concentrations are lower in GH-transgenic carp. *Agrp1* (agouti-related protein 1) and *sla* (somatolactin *a*), which are related to appetite and lipid catabolism, are significantly higher in GH-transgenic carp. Low glucose content and increased appetite indicate disrupted metabolic and energy deprivation status in GH-transgenic carp. Meanwhile, the expression of genes, such as *gnrhr2* (gonadotropin-releasing hormone receptor 2), *gthα* (gonadotropin hormone, alpha polypeptide), *fshβ* (follicle stimulating hormone, beta polypeptide), *lhβ* [luteinizing hormone, beta polypeptide] in the pituitary, *cyp19a1a* (aromatase A) in the gonad, and *cyp19a1b* (aromatase B) in the hypothalamus, are decreased in GH-transgenic carp. In contrast, pituitary *gnih* (gonadotropin inhibitory hormone), *drd1* (dopamine receptor D1), *drd3* (dopamine receptor D3), and *drd4* (dopamine receptor D4) exhibit increased expression, which were associated with the retarded reproductive development. Leptin receptor mRNA was detected by fluorescence *in situ* hybridization in the pituitary including the pars intermedia and proximal pars distalis, suggesting a direct effect of leptin on LH. Recombinant carp Leptin protein was shown to stimulate pituitary *gthα, fshβ, lhβ* expression, and ovarian germinal vesicle breakdown *in vitro*. In addition to neuroendocrine factors, we suggest that reduced hepatic leptin signaling to the pituitary might be part of the response to overexpression of GH and the resulting delay in puberty onset.

## Introduction

Growth and reproduction are closely related, and there is cross talk between the endocrine systems controlling these fundamental processes in vertebrates (1–3). Examples of the clinical manifestations of the growth–reproduction interaction include delayed onset of puberty and ovarian problems in women with growth hormone (GH) insufficiencies (4). GH hypersecretion in acromegaly can also be associated with menstrual disturbances and reduced fertility (5). In normal animals, GH can stimulate testicular spermatogenesis and ovarian hormone synthesis (6, 7) or indirectly affect gonad development by stimulating the expression of insulin-like growth factor 1 (IGF1) (8). As GH participates in hormone synthesis, ovulation, growth and renewal of follicles, oocyte maturation, spermatogenesis, sperm motility, and other aspects of reproductive development, it may be considered as a co-gonadotropin (9).

Paradoxically, reproduction in GH-transgenic fish is often reduced or disrupted to some extent. Stable GH-transgenic fish lines, including tilapia (*Oreochromis niloticus*) (10), coho salmon (*Oncorhynchus kisutch*) (11), loach (*Misgurnus mizolepis*) (12), and common carp (*Cyprinus carpio*) (13) have been established as fast-growing genetically modified organisms for potential human consumption. After decades of controversy, in 2015 the Federal Department of Agriculture in the US approved the first GH-transgenic Atlantic salmon (*Salmo salar*) as a legally edible animal. This is a milestone for transgenic animal industrialization, yet challenges remain. Beside increased growth, GH-transgenic fish species exhibit reduced courtship and spawning (14, 15), reduced sperm quantity and ovarian size (10, 16), and reduced nest loyalty, quivering frequency, and spawning participation (17).

Rahman (10) and Bessy (14) were among the first to report increased energy allocation to somatic growth rather than gonad development for reduced gonadal size and reproductive potential in GH-transgenic fish. We also reported on delayed sexual maturation and decreased gonadal size of fast-growing GH-transgenic common carp (18). In that study, high levels of GH were shown to directly inhibit luteinizing hormone (LH) production and release through GH receptors in pituitary gonadotrophs to suppress reproductive processes. This provided the first proposal of a mechanism whereby GH overexpression in transgenic fish could affect reproduction. However, the exact mechanism linking altered energy allocation, somatic growth, and suppressed reproduction in GH-transgenic animals remains to be elucidated.

The present study was therefore conducted using RNA sequencing of hypothalamus, pituitary, gonad, liver from 5-month-old GH-transgenic common carp, and their wild-type counterparts. An association between decreased *leptin* expression and the overexpression of GH in the transgenic animals led us to investigate the role of leptin. This is especially relevant given that leptin plays critical roles in the regulation of body weight by inhibiting food intake and stimulating energy expenditure. We determined that recombinant carp Leptin directly stimulates both pituitary gonadotropin subunit expression and ovarian germinal vesicle breakdown (GVBD). In GH-transgenic common carp, decreased *leptin* expression is therefore one link between altered energy status and disrupted reproduction.

## Materials and Methods

### Experimental Fish

The female and male GH-transgenic common carp (Yellow river strain) used in the study were from our TG2 line, carrying the grass carp (*Ctenopharyngodon idellus*) GH gene (13). GH-transgenic and non-transgenic common carp were derived from the same non-transgenic mother and were reared at Guanqiao Experimental Depot, Wuhan, China. Ten individuals of each group were sampled, and the body weights were measured. Five fish were sampled in each group for RNA sequencing. Ten fish were sampled for qPCR and serum hormone analysis. The gonadosomatic index (GSI; gonad weight/body weight × 100%) was also calculated for each individual. The Animal Care and Use Committee of the Institute of Hydrobiology approved all procedures.

### RNA Isolation, Library Construction, and Sequencing

Tissue samples of the hypothalamus, pituitary, liver, and gonad were collected from five GH-transgenic and non-transgenic common carp in 5-month-old at puberty developmental stage, respectively. Total RNA was isolated using the Trizol reagent (Invitrogen, USA), according to the manufacturer’s protocol. All of the samples had an RNA integrity number value greater than 8. Sequencing libraries were generated using the NEB Next Ultra RNA library prep kit from Illumina (New England Biolabs, USA), according to the manufacturer’s protocol. Libraries were sequenced on an IlluminaHiseq™ 2000 platform and 150 bp paired-end reads were generated. The transcriptome raw data are available at http://www.ncbi.nlm.nih.gov/bioproject/337990.

### Data Analysis

Clean data (clean reads) were obtained by removing reads with adaptors, reads with unknown sequence more than 5% or low quality reads which had more than 50% QA ≤ 15 bases, by using in-house Perl scripts. All further analyses were performed using only the cleaned, high-quality data. *De novo* transcriptome assembly was carried out using the Trinity program (19) with optimized k-mer length of 25. On the other hand, the clean reads were also mapped to the common carp (Heilongjiang strain) reference mRNA database^1^ using SOAPaligner/soap2. Two base mismatches were allowed in the mapping process, total mapped reads were calculated, and the mapped regions were counted. HTSeq software was used to count the number of reads mapped to each gene. The normalized gene expression level was separately calculated as reads per kilobase of mRNA per million of mapped reads (RPKM) for each library.

The transcriptomic data included expressed genes in hypothalamus, pituitary, gonad, and liver of both female and male individuals of GH-transgenic and non-transgenic carp. There were in total 16 libraries constructed and sequenced. The mixed library was constructed following *de novo* assembly processes, including 172,823 unigenes, which was far more than the estimated gene number of common carp genome. The clean reads were also mapped to the reference mRNA database of common carp. The average percentage of total mapped reads was 38.09% and that of unique mapped reads was 32.71% (Tables S1 and S2 in Supplementary Material). The common carp database was compared to the *de novo* assembled reference database using the Basic Local Alignment Search Tool.^2^ We then assembled a new reference database that included unique sequences of Yellow River common carp strain and excluded any redundancies. This improved database containing 52,327 genes was used as the reference database for further analysis. The average percentage of total mapped reads was improved to 53.83% and that of unique mapped reads was improved to 47.55% (Table S3 in Supplementary Material). The following differential expression analysis was based on the improved reference database.

### Differential Gene Expression Analysis

The expression level of each gene was estimated by RPKM values. The 16 libraries were named NTHF, NTHM, THF, THM, NTPF, NTPM, TPF, TPM, NTLF, NTLM, TLF, TLM, NTGF, NTGM, TGF, and TGM where NT and T indicates non-transgenic (NT) or transgenic (T) groups. The four tissues were hypothalamus (H), pituitary (P), liver (L), and gonads (G) in female (F) and male (M) common carp. Data sets were subjected to a series of comparisons between GH-transgenic and non-transgenic groups. The number of differentially expressed genes (DEGs) identified is listed in Table S4 in Supplementary Material. Detailed information of all DEGs is shown in Table S5 in Supplementary Material. Since we focused on the delayed gonadal development in both female and male GH-transgenic carp, the genes changed both in female and male individuals were filtered using Venn diagram analysis (Figure S1 in Supplementary Material). Detailed information of DEGs in both male and female of different tissues is shown in Table S6 in Supplementary Material.

Differential expression analysis was performed using the DESeq package (20). The resulting *P*-values were adjusted using Benjamini and Hochberg’s method for controlling the false discovery rate. Genes with an adjusted *P*-value less than 0.05 were considered to be differentially expressed. Gene ontology (GO) enrichment analysis of DEGs was implemented by the GOseqR package (21) with gene length bias correction. For hypothalamus of GH-transgenic carp and non-transgenic carp, annotated genes were categorized into cellular component and molecular function (Figure S2 in Supplementary Material). Pituitary DEGs were categorized into molecular function categories. In the liver of GH-transgenic and non-transgenic carps, the DEGs were categorized into cellular component, molecular function, and biological processes. In gonad, the DEGs were categorized into cell component change. The detailed list is attached in Table S7 in Supplementary Material.

The KOBAS software (22) was employed to test the statistical enrichment of DEGs in Kyoto Encyclopedia of Genes and Genomes database (KEGG^3^) pathways. The KEGG terms with corrected *P*-values less than 0.05 were considered significantly enriched. In order to identify possible biochemical pathways that DEGs were involved in, KEGG analysis was carried out to understand the common changed pathways that enriched in both male and female GH-transgenic carp. There are 81 significantly enriched terms in hypothalamus, pituitary, liver, and gonad which are listed in Table S8 in Supplementary Material.

To further investigate reproductive development in GH-transgenic carp, significant DEGs in tissues common to both females and males were identified and annotated. We filtered those DEGs exhibiting a log_2_ fold change ≥ 1 to include those with a RPKM value higher than 10 in at least one sample and those genes reported to be involved in either endocrine control of gonadal development or energy regulation. These DEGs are listed in Table S8 in Supplementary Material.

### Validation of DEGs by qPCR

In order to confirm the reliability of the data obtained by RNA-seq, 15 DEGs were selected for validation using qPCR. The primers are listed in Table S9 in Supplementary Material. The RNA samples from an independently repeated study were used for reverse transcription. qPCR was carried out on a Bio-Rad fluorescence quantitative PCR instrument (CFX96 Touch™). Each qPCR mixture contained 0.8 µL sense and reverse primers, 1 μL template, 10 µL 2 × SYBR mix (TOYOBO, Japan), and 7.4 µL ddH_2_O. Three replicates were conducted for each sample, and β-actin gene was used as an internal control. The program for qPCR was as follows: 95°C for 10 s, 40 cycles of 95°C for 5 s and 60°C for 20 s. Relative expression level was calculated using the 2^−ΔCt^ method. All data are given as mean ± SD of three replicates.

### *In Situ* Hybridization and Immunofluorescence

The leptin receptor *(lepR)* probes for *in situ* hybridization were made using the primer pair (5′-TTATCTAATCATCCAGTGC-3′; and 5′-TAATACGACTCACTATAGGGCGGAGAACGGTCGAGTA-3′), following validated protocols (23). *In situ* hybridization for *lepR* combined with immunocytochemical localization of Lhβ (antibody FMU-cGTHIIβ9) in non-transgenic female common carp (8 months old) was performed as previously described (18).

### Determination of Serum Hormone Concentrations

Blood was collected from GH-transgenic and non-transgenic common carp (5 months old, *n* = 7–15). Serum samples were obtained by centrifugation at 3,000 × *g* for 15 min at 4°C. The Gh ELISA system, with the assay range of 1.56–50 ng/mL, was developed and validated in our previous studies (24). The estradiol ELISA kit (#582251) was purchased from Cayman Chemical (USA). Igf1 (CSB-E12122Fh) and Igf2 (CSB-EL011088FI) ELISA kits were purchased from Cusabio Life Science (Wuhan, China) (25). The hormone analyses followed the manufacturer’s instructions.

### *In Vitro* Incubation with Recombinant Leptin

Full-length cDNA of common carp *leptin* was inserted into the pMXB10b vector (New England Biolabs, Beijing, China), and the recombinant construct was transformed into DE3 competent cells. The leptin protein fused to a chitin binding domain was expressed in the bacteria following induction with IPTG. After incubation with 0.3 mM IPTG at 30°C for 6 h, the bacteria were harvested and disrupted by sonication (Scientz-IID, Ningbo, China). The bacterial lysate was passed through a chitin column, and the anchored fusion protein was then incubation with 50 mM DTT, to cleave and harvest the recombinant common carp Leptin protein. The purity of the recombinant protein was confirmed by polyacrylamide gel electrophoresis (Figure S3 in Supplementary Material).

Pituitary glands were removed from non-transgenic common carp (5 months old). After being washed three times, the pituitaries were cut into small pieces with scissors and equally transferred into 24-well plates for the following incubation. To determine the effects of Leptin on pituitary *gthα, fshβ*, and *lhβ* gene expression, carp pituitary fragments were treated with recombinant Leptin. The dosage of Leptin was chosen according to serum Leptin concentration (~10^−9^ M) we determined in this study and reported in other animals (26–28). Pituitary fragments were harvested at 30 min, 1 h, and 2 h after incubation. Levels of *gthα, fshβ*, and *lhβ* were quantified by real-time PCR. Data presented in this study were the results of at least three independent experiments and were expressed as fold change relative to the controls.

### GVBD Assay

Zebrafish ovarian follicles were isolated and incubated following an established protocol (29). Briefly, gravid female zebrafish were deeply anesthetized with 0.01% tricaine methanesulfonate solution (Sigma, USA) for 2 min and sacrificed. Ovaries were washed three times in PBS, and the individual ovarian follicles were separated without damaging the follicle cell layers. Full-grown follicles (550–650 µm in diameter) were selected and randomly distributed into a 24-well plate (20–30 follicles per well) and treated with recombinant Leptin protein. The follicles were incubated for 4–16 h and scored at each time point for %GVBD.

## Results

### Body Weights and GSI in GH-Transgenic Carp

**Figure 1:**
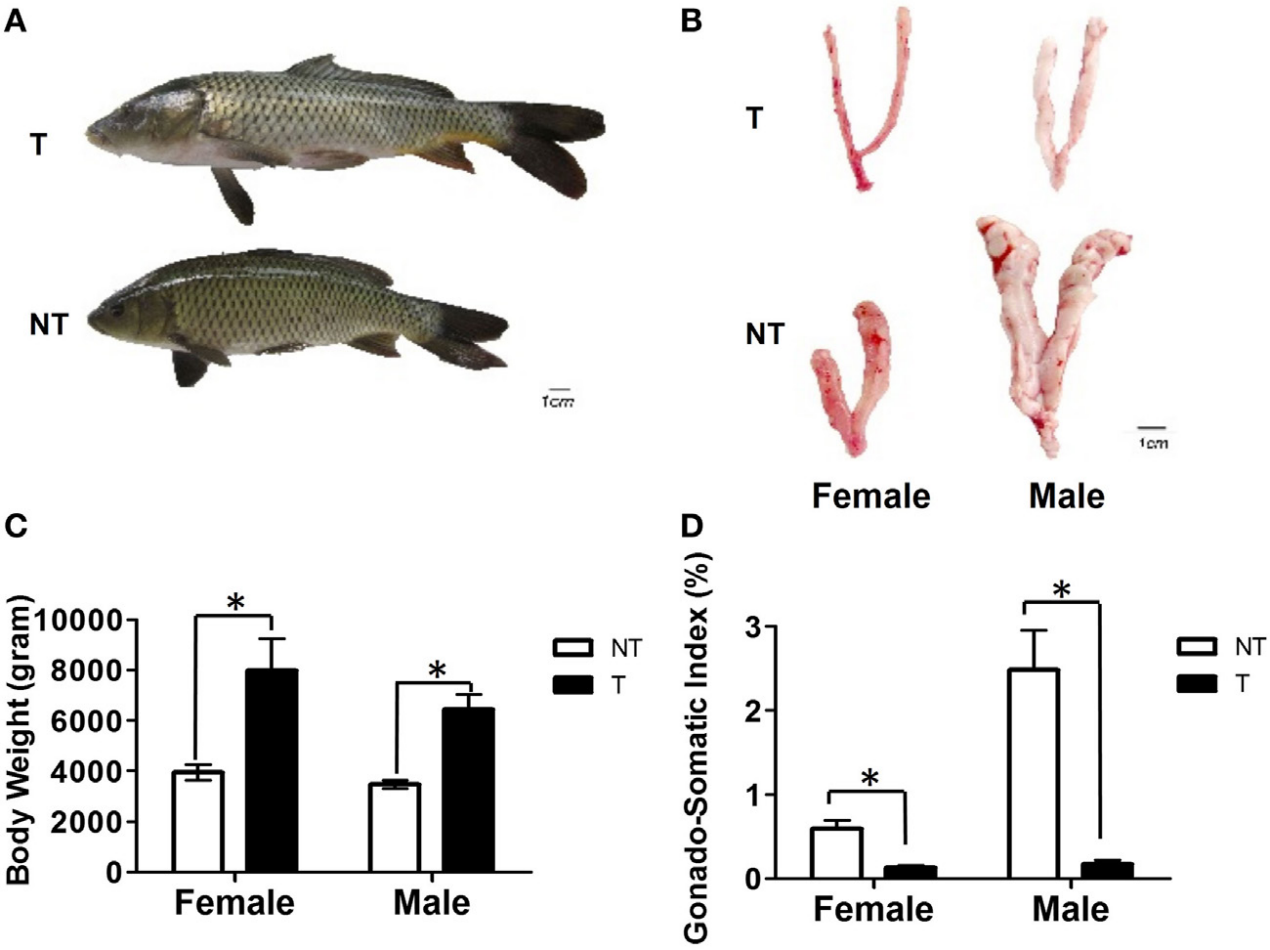
Comparison of body growth and gonadal development between non-transgenic (NT) and GH-transgenic (T) common carp of 5 months. **(A)** Comparison of morphology of NT and T common carp. **(B)** Comparison of the gonad morphology of NT and T common carp. **(C)** Comparison of body weight of female and male NT and T common carp. **(D)** Comparison of gonadal-somatic index between NT and T common carp of females and males. Samples were collected at 5 months of age. Values are represented as means ± SEM (*n* = 10–15) at each sampling time and analyzed by Student’s t-test between NT and T. Asterisks indicate statistically significant differences compared within NT and T at *P* < 0.05.

The body weights of GH-transgenic carp were 1.8- and 1.9-fold higher than female and male non-transgenic animals, respectively (Figures 1A,C). The GSI in GH-transgenic carp decreased by 2.5- and 9.8-fold compared with non-transgenic carp (Figures 1B,D).

### Differential Gene Expression in GH-Transgenic Carp

Analysis of the DEGs in hypothalamus revealed that four terms involved in signal transduction and signaling molecules and interaction were enriched. These included neuroactive ligand receptor interaction, the Jak-STAT signaling pathway, cytokine–cytokine receptor interaction, and cell adhesion molecules (Table S7 in Supplementary Material). The gene with the highest expression (with the highest RPKM value) in wild-type is *cyp19a1b*, or aromatase b, which is the estrogen synthesis enzyme found in radial glial cells in the teleost brain. Expression of *cyp19a1b* was downregulated more than twofold (with log_2_Ratio (T/NT) −1.2 for female and −1.3 for male common carp, Table S8 in Supplementary Material) in the hypothalamus of GH-transgenic carp. Hypothalamic *slα* (somatolactin alpha) was also decreased in GH-transgenic fish. Genes that were upregulated in the hypothalamus of GH-transgenic carp include the growth-related transcriptional factor *stat1* (signal transducer and activator of transcription 1), *stat2* (signal transducer and activator of transcription 2), and *irs2* (insulin receptor substrate 2) (Table S8 in Supplementary Material).

**Figure 2:**
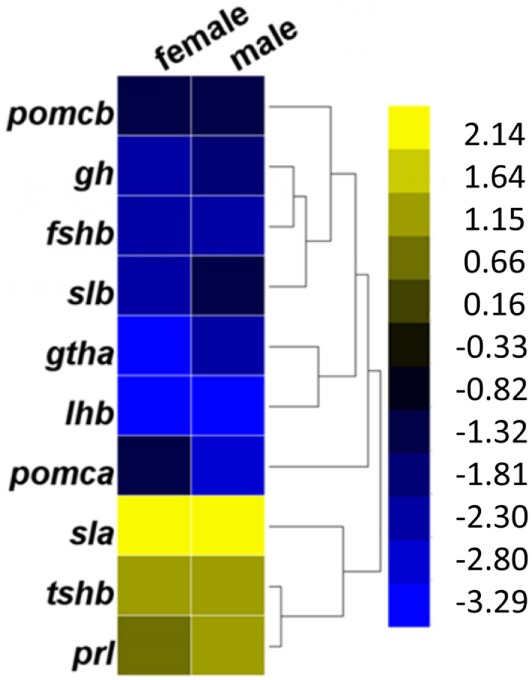
Changed expression pattern of genes in GH-transgenic (T) common carp compared with non-transgenic (NT) common carp. Fold change of pituitary hormone-encoded genes in female and male T common carp compared with NT animals. Yellow represented for upregulation, dark blue and dark yellow for nearly no expression change, and blue for downregulation.

In the pituitaries of GH-transgenic carp, the expression levels of genes encoding hormones such as *gh, gthα, fshβ*, and *lh* β exhibited downregulation, while *prl* (prolactin) was upregulated (Figure 2). These genes are related to the GO terms neuroactive ligand–receptor interaction pathway and endocrine system. Growth-related genes in the pituitary that were differentially expressed in GH-transgenic carp include upregulated *slα, tshr* (thyroid stimulating hormone receptor), *sstr2* (somatostatin receptor 2), *sstr3* (somatostatin receptor 3), *socs1* (suppressor of cytokine signaling 1), *socs3b* (suppressor of cytokine signaling 3b), *socs5a1* (suppressor of cytokine signaling 5a1), *socs5* (suppressor of cytokine signaling 5), *slα, irs2* (insulin receptor substrate 2), *sst* (somatostatin), *lepR*, and *lepRl* (leptin receptor long isoform), and downregulated *gh* and *igfbp1* (insulin-like growth factor binding protein 1) (Table S8 in Supplementary Material). These genes are related to the Jak-STAT signaling and cytokine–cytokine receptor interaction signaling pathways. In the pituitary, reproduction-promoting genes such as *gnrhr2* (gonadotropin-releasing hormone receptor 2), *fshβ, lhβ, cyp19a1b*, and *gabar* (gamma-aminobutyric acid receptor-associated protein-like) were downregulated, while reproduction-inhibiting genes such as *drd1* (dopamine receptor D1) and *drd3* (dopamine receptor D3) were upregulated in GH-transgenic carp. Genes related to circadian rhythms such as *clock1a, clock2*, and *per2* were upregulated while *cyclinG2* was downregulated in the pituitaries of GH-transgenic carp. Markers for reactive oxygen species such as *ho* (heme oxygenase 1) and *grik4* (glutamate receptor ionotropic, kainate 4) were also upregulated in the GH-transgenic group.

In the liver, most DEGs were related to the GO terms of lipid metabolism, amino acid metabolism, and carbohydrate metabolism (Table S7 in Supplementary Material). We found that the hepatic expression levels of *erβ1* (estrogen receptor beta1), *ho, desaturase*, and *fabp7* (fatty acid binding protein 7) were increased in GH-transgenic carp while those of *leptinI, ghrelin, epo* (erythropoietin), *gys, igfbp1* decreased (Table S8 in Supplementary Material). All these genes are associated with the lipid metabolism signaling pathway, which is also related to reproductive development.

In the gonad, DEGs in both male and female were associated with organismal systems including the immune system, development, endocrine system, and digestive system. For specific genes related to reproductive and gonadal development, *cystatin* and *zp3* expression were upregulated, whereas *trypsin, npsn* (nephrosin precursor), *igf3*, and *cyp19a1a* expression were down-regulated in the GH-transgenic carp gonads (Table S8 in Supplementary Material).

### Validation of RNA-Seq Data

To validate the RNA-Seq data, 18 DEGs were selected for qPCR analysis. These genes included *fshβ, lhβ, gthα, gh, mch* (melanin-concentrating hormone), *gnih, pkm* (pyruvate kinase), *gnrh3, drd1, drd2, drd3, drd4, gnrhr1, gnrh*r*2, cyp19a1a, cyp19a1b, igf3*, and *leptinI* (Table S10 in Supplementary Material). As shown in Table S10 in Supplementary Material, the direction of change in expression of all 18 DEGs as determined by qPCR was consistent with the RNA-seq results. The Pearson product-moment correlation coefficient for results obtained from qPCR and RNA-seq for the 18 genes in both sexes was *R* = 0.835 (*P* < 0.0001), which confirmed the reliability and accuracy of the RNA-seq data.

**Figure 3:**
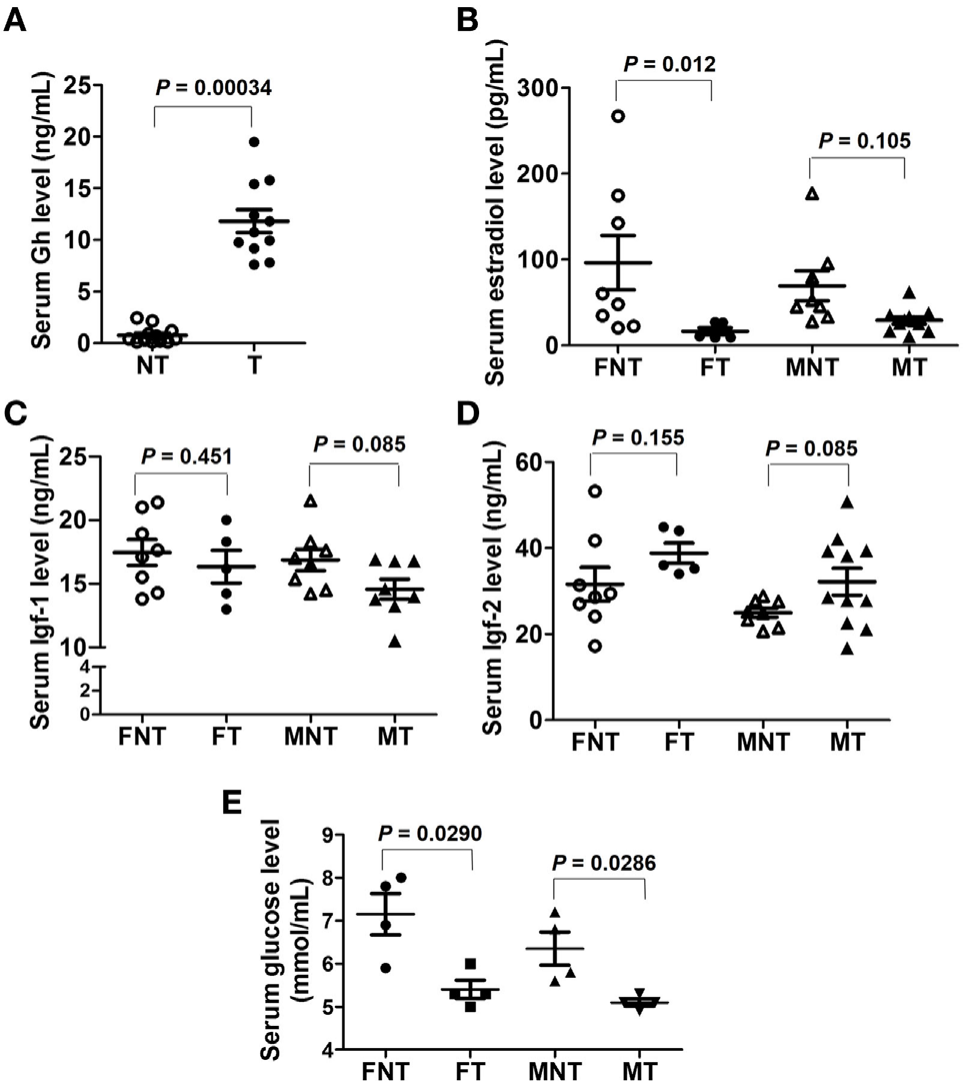
Serum levels of **(A)** Gh, **(B)** estradiol, **(C)** Igf1, **(D)** Igf2, and **(E)** glucose contents in female (F) and male (M) non-transgenic (NT) and GH-transgenic (T) common carp. The results obtained were analyzed within sex by independent sample *t*-test. The *P*-values are indicated on the graph.

The levels of Gh, estradiol, Igf1, and Igf2 were quantified using ELISA. The results showed that serum Gh levels were significantly increased in GH-transgenic carp (Figure 3A). Serum estradiol levels were lower in GH-transgenic female carp, while there were no effects of GH-transgenesis in males (Figure 3B). There was no difference in Igf1 and Igf2 levels in GH-transgenic carp compared with non-transgenics (*P* > 0.05) (Figures 3C,D). Serum glucose levels were decreased in both female and male GH-transgenic carp compared with non-transgenic animals (Figure 3E). As determined using Western blotting, pituitary Gh, Lhβ, and Gthα subunit levels were lower in GH-transgenic carp (Figure S4A in Supplementary Material), whereas Prl levels were higher in GH-transgenic carp than non-transgenic carp (Figure S4A in Supplementary Material). Semi-quantitative PCR showed that *gnrhr1* was expressed mainly in hypothalamus, whereas *gnrhr2, gnrhr3*, and *gnrhr4* mainly in pituitary (Figure S4B in Supplementary Material). These results also support the reliability of the RNA-seq data.

### Effects of Recombinant Leptin on Pituitary Gonadotropin Subunit Expression and Ovarian Follicular Maturation *In Vitro*

**Figure 4:**
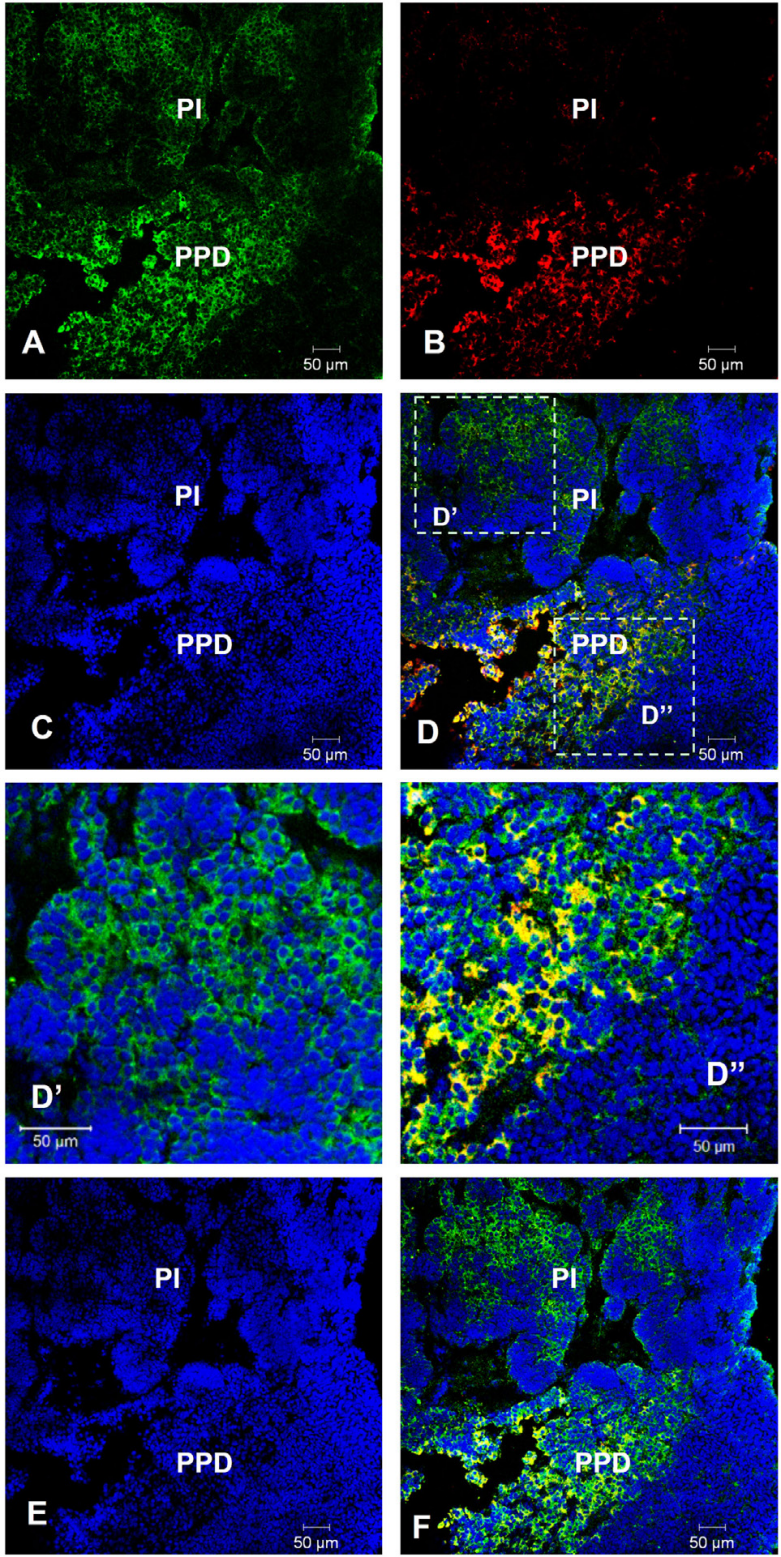
Co-localization of *lepR* and Lh in non-transgenic common carp pituitary. *In situ* hybridization of *lepR* (green) and immunofluorescence of Lh (red) in 8-month-old female **(A–D,D′,D″)** and male **(E,F)** common carp pituitary. Yellow-orange color indicates colocalization. PI, pars intermedia; PPD, proximal pars distalis. Scale bar, 50 µm.

We found that *lepR* is expressed in the pituitary and is upregulated in GH-transgenic carp (Table S10 in Supplementary Material). Given its importance in both body weight regulation and reproduction (30, 31), we therefore focused our attention on leptin. The *lepR* mRNA was detected by fluorescent *in situ* hybridization in the pars intermedia and proximal pars distalis (PPD) of the female pituitary. The *lepR* signal in PPD was co-localized with Lhβ (Figure 4).

**Figure 5:**
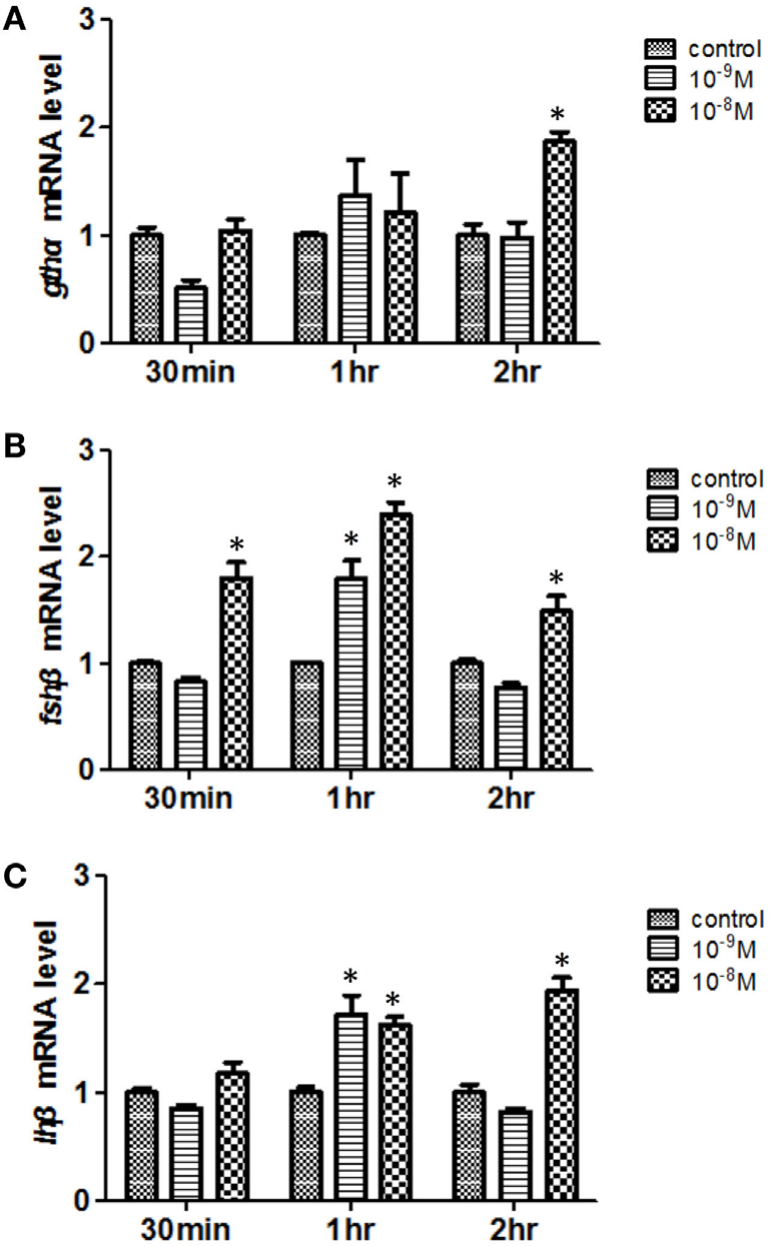
Effects of Leptin on **(A)** *gthα*, **(B)** *fshβ*, and **(C)** *lhβ* expression in 5-month-old female non-transgenic carp pituitary cells *in vitro*. Pituitary cells were exposed to Leptin for 30 min, 1, and 2 h. The expression of the target genes (mean ± SEM) were determined by real-time PCR and were expressed as fold change normalized to the controls. The results obtained were analyzed by independent sample *t*-test compared with controls. Asterisks indicate statistically significant differences at *P* < 0.05.

To further study whether there is a direct effect of Leptin on reproductive processes, we first tested the effects of the recombinant common carp Leptin protein on pituitary *gthα, fshβ*, and *lhβ* expression using real-time PCR (Figure 5). Two-way ANOVA was used to determine the concentration and time-dependent effects of Leptin on expression of the gonadotropin subunits. For *gthα*, the concentration × time interaction was significant (*P* = 0.01). While the main effects of increasing Leptin on *gthα* were not statistically significant (*P* = 0.22), there was an overall increase in expression of *gthα*, with time (*P* = 0.009). Only at the concentration of 10^−8^ M did Leptin increase (*P* < 0.05) *gthα* at 2 h (Figure 5A). For *fshβ*, the concentration × time interaction was significant (*P* = 0.01). Levels of *fshβ* increased with time (*P* = 0.0002) and with concentration of leptin (*P* < 0.0001). The 10^−9^ M dose increased *fshβ* up to 1 h of incubation. Leptin concentrations of 10^−8^ M were the most effective at enhancing *fshβ* from 30 min to 2 h of incubation (Figure 5B). The overall pattern of expression of *lhβ* resembled that of *fshβ*. For *lhβ*, the concentration × time interaction was significant (*P* = 0.0007). Levels of *lhβ* increased with time (*P* = 0.0018) and concentration of Leptin (*P* < 0.0017). The 10^−9^ M dose of Leptin increased *lhβ* only at 1 h of incubation. For 10^−8^ M Leptin, *lhβ* was increased at both 1 and 2 h of incubation (Figure 5C).

**Figure 6:**
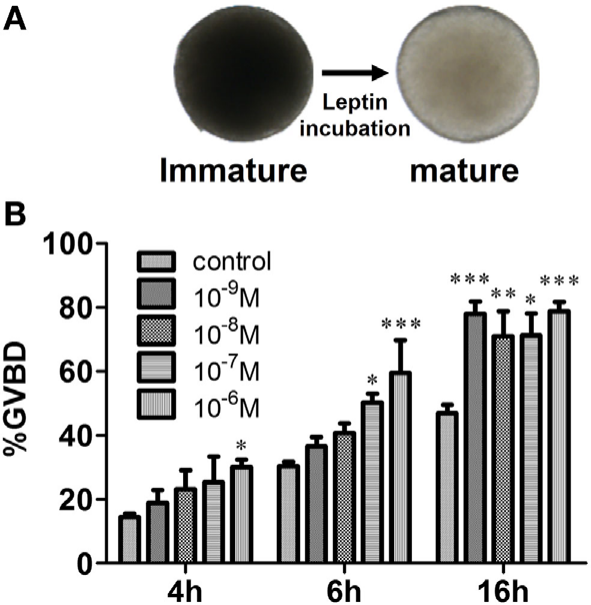
Effects of recombinant carp Leptin on zebrafish oocyte maturation. **(A)** Fully growth follicles were treated with recombinant carp Leptin. The follicles became mature (transparent) after germinal vesicle breakdown (GVBD). **(B)** The % follicles undergoing GVBD was scored and assessed. Each value represents the mean ± SEM of three independent experiments.

Recombinant common carp Leptin also stimulated zebrafish follicle maturation as assessed using the *in vitro* GVBD assay (Figure 6). Two-way ANOVA was used to determine the concentration and time-dependent effects of Leptin on GVBD. Both time (*P* < 0.0001) and Leptin concentration (*P* < 0.0001) increased GVBD over the 4–16 h incubation period. Although the interaction of time × concentration was not significant (*P* = 0.26), lower Leptin concentrations were less effective over short incubation times, and all doses of Leptin enhanced GVBD at 16 h.

## Discussion

In the present study, we uncovered a novel mechanism for energy allocation between growth and reproduction in GH-transgenic fish. Common carp, tilapia, and coho salmon that carry a GH-transgene have enhanced growth and delayed reproductive development (10, 14, 18). Such genetically modified fish are amenable models to study interactions between the endocrine axes controlling growth and reproduction. Unfortunately, most published studies have only speculated that increased energy allocation to somatic growth rather gonad development is the reason for reduced reproductive performance in transgenic fish (10, 14). Our data indicate that in addition to neuroendocrine factors, reduced hepatic leptin may be part of the response to overexpression of GH and the resulting delayed in puberty onset.

### Leptin May Act as a Sensor for Energy Status to Regulate Reproductive Development in GH-Transgenic Common Carp

The DEGs in GH-transgenic compared to non-transgenic common carp were related to feeding, growth, metabolism of glucose and lipids, and reproductive function. Growth-enhanced GH-transgenic common carp exhibit increased appetite, which was previously surmised to be driven at least partially by increased *AgRP1* expression (32). In this study, we found the reduced expression of *gys* (glycogen synthase) and *igfbp1*, which was accompanied by decreased blood glucose level in GH-transgenic carp. Improved appetite in GH-transgenic carp could be driven by low glucose (33–35). On the other hand, decreased fat and energy content (32, 36), together with improved appetite and metabolic rate (32, 37, 38) in GH-transgenic fish, reflects an energy-deprived status. Under low glucose conditions, lipids can be used as energy sources. We observed that the lipid metabolism pathway was activated in the livers of GH-transgenic common carp. In particular, elevated pituitary *slα* in GH-transgenic carp may be important because somatolactin stimulates lipid catabolism in fish (39, 40). Increased hepatic *fas* (fatty acid synthase) expression in the GH-transgenic carp could also be indicative of accelerated fatty acid synthesis.

Notably, GH-transgenic common carp exhibit decreased hepatic *leptin* expression level, positively related to decreased fat content, as previously reported (41). Thus, leptin might play an important role in energy sensing. On the other hand, leptin is suspected to have a role in reproductive development in teleost fish. In sexual mature Atlantic salmon, the expression level of *leptin* is higher than in immature animals (31). In mammals, it is known that leptin affects both pituitary and gonadal functions (42). In addition, the effect of leptin on the activation of GnRH neurons is suspected to be indirect (43). We found that levels of pituitary *gthα, fshβ*, and *lhβ* mRNAs were stimulated by Leptin *in vitro*. We show that carp LH cells express the leptin receptor as reported in other fish (44, 45). Moreover, we demonstrate that recombinant Leptin stimulates GVBD in zebrafish. Together these results indicate that a GH–leptin–gonadotropin axis at least partially mediates the cross talk between growth and reproduction in fish.

### Other Factors Are Linked to Reduced Reproductive Performance in GH-Transgenic Common Carp

Our previous study reported that elevated Gh production in GH-transgenic common carp suppressed pituitary Lh content and serum Lh levels (18). Zhou et al. (46) first demonstrated the intrapituitary autocrine/paracrine regulation between gonadotrophs and somatotrophs in grass carp pituitary cells and found that Gh inhibits Lh secretion *in vitro*. *In vivo* in the GH-transgenic carp model, GH regulates reproductive development through paracrine effects whereby Gh inhibits pituitary *gthα, fshβ*, and *lhβ* expression and Lh secretion (18).

Our data suggest that Gh may also negatively regulate reproductive processes at other levels of the hypothalamic–pituitary–gonadal axis. In cyprinid fish, GnRH3 is the main form stimulating Lh release (47). In the present study, the expression of *gnrh3* did not change in GH-transgenic common carp, while the expression of *gnrhr2*, the mRNA encoding for the GnRH receptor mediating the effects of GnRH3 in the pituitary, decreased significantly. Previously, we demonstrated that GH-transgenic carp have reduced pituitary sensitivity because coinjection of a GnRH agonist and dopamine agonist was not very effective at increasing *lhβ* and *fshβ* expression in GH-transgenic versus wild-type carp (18). Thus, the downregulation of *gnrhr2* caused by Gh is a contributing factor to reduced gonadotropin production. RNA sequencing and targeted PCR indicate that expression of several dopamine receptor subtypes were increased in the GH-transgenic carp. Increased expression of *drd1, drd3*, and *drd4* was confirmed by PCR. It has long been known that in goldfish (*Carassius auratus*), DA neurons innervate the anterior pituitary and stimulate Gh secretion *via* D1 receptors (48). It is unknown how Gh may regulate *drd1* and how it may relate to decreased expression of endogenous pituitary Gh noted in this study of GH-transgenic carp. On the other hand, there is clear evidence that DA acting *via* the pituitary DA D2 receptor is a critical inhibitor of gonadotropin synthesis and secretion in many teleosts (49, 50). Therefore, it is important to note that expression of *drd2* was not significantly different (only 1.2-fold) in the pituitaries of GH-transgenics. On the other hand, *drd3* and *drd4b* are members of the D2-like family, and they were significantly upregulated. A lesser known inhibitory effect of DA *via* D1 receptors has been reported for goldfish (51), such that a DA D1 antagonist potentiates glutamatergic stimulation of Lh release. Moreover, DA D3- and D4-selective drugs can inhibit GnRH-stimulated LH release from cultured tilapia pituitary cells (52). Perhaps, Gh also enhances dopaminergic inhibition of LH in GH-transgenic carp by increased DA receptor expression. Increased expression of hypothalamic *gnih* was accompanied by increased *gnihr* in pituitary in GH-transgenic common carp. In reproductively active adult goldfish (53) and carp (54), GnIH-3 is a strong inhibitor of LH.

The suppressed expression of gonadotropin subunits in the pituitary and decreased Lh and Fsh concentrations on the blood were accompanied by significantly reduced blood estradiol in females and a tendency for the same in males. It is known that physiological concentrations of estradiol exert strong positive feedback effects at the pituitary to enhance GnRH-induced Lh secretion in goldfish of both sexes with intact gonads (2, 55). The decrease in estradiol may therefore result in a reduced stimulation of *gthα, fshβ*, and *lhβ*.

The expression of *igf*3 in the female and male gonads decreased significantly, which might also be related to the delayed gonadal development in GH-transgenic carp. Igf3 is a gonadal insulin-like growth factor expressed specifically in follicular and Sertoli cells (56, 57). It functions to promote oocyte maturation in females (58) as well as spermatogonial proliferation in the testes (59).

## Conclusion

**Figure 7:**
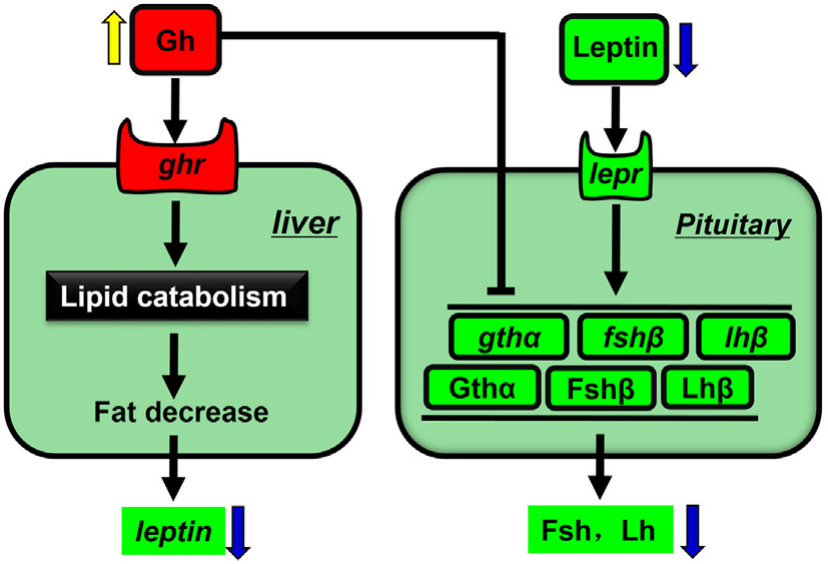
Simplified model for the influence of growth hormone (GH) overexpression on disruption of reproduction in common carp. Genes and proteins that are upregulated in GH-transgenic carp are shown in red, while those downregulated are shown in green. Black arrows indicate stimulation, and black blunt-end lines indicate suppression.

Overexpression of Gh leads to increased somatic growth and altered glucose and lipid metabolism in common carp. We observed that hepatic leptin, pituitary gonadotropin subunit expression was decreased in GH-transgenic carp with reduced gonadal growth. This led us to explore the link between reduced leptin and suppressed reproductive processes. We found that recombinant carp Leptin stimulates gonadotropin subunit expression and induced ovarian GVBD *in vitro*. This provides evidence for a GH–leptin–gonadotropin axis that may mediate the cross talk between growth and reproduction in GH-transgenic fish (Figure 7).

## Author Contributions

Conceived and designed the experiments: WH, MC, and ZZ. Performed the experiments: JC and MC. Analyzed the data and interpreted the results: MC, JC, AZ, and MS. Contributed reagents/materials/analysis tools: WH, BT, YL, and YW. Wrote and revised the paper: JC, MC, VT, and WH.

## Conflict of Interest Statement

The authors declare that the research was conducted in the absence of any commercial or financial relationships that could be construed as a potential conflict of interest.

## Footnotes

^1^http://www.carpbase.org (Accessed: September 27, 2015).

^2^https://blast.ncbi.nlm.nih.gov/Blast.cgi (Accessed: October 08, 2015).

^3^http://www.kegg.jp/ (Accessed: November 15, 2015).

## References

[1] Le Gac F Blaise O Fostier A Le Bail PY Loir M Mourot B Growth hormone (GH) and reproduction: a review. Fish Physiol Biochem (1993) 11:219–32. 10.1007/BF00004569 24202479

[2] Trudeau VL. Neuroendocrine regulation of gonadotrophin II release and gonadal growth in the goldfish, *Carassius auratus*. Rev Reprod (1997) 2:55–68. 10.1530/ror.0.0020055 9414466

[3] Hull KL Harvey S. Growth hormone and reproduction: a review of endocrine and autocrine/paracrine interactions. Int J Endocrinol (2014) 2014:234014. 10.1155/2014/234014 25580121 PMC4279787

[4] Spiliotis BE. Growth hormone insufficiency and its impact on ovarian function. Ann N Y Acad Sci (2003) 997:77–84. 10.1196/annals.1290.009 14644812

[5] Herman-Bonert V Melmed S Pregnancy and acromegaly. In: Bronstein MD, editor. Pituitary Tumors in Pregnancy. Massachusetts: Kluwer Academic Publishers (2001). p. 109–21.

[6] Van Der Kraak G Rosenblum PM Peter RE. Growth hormone-dependent potentiation of gonadotropin-stimulated steroid production by ovarian follicles of the goldfish. Gen Comp Endocrinol (1990) 79:233–9. 10.1016/0016-6480(90)90108-X 1697273

[7] Miura C Shimizu Y Uehara M Ozaki Y Young G Miura T. Gh is produced by the testis of Japanese eel and stimulates proliferation of spermatogonia. Reproduction (2011) 142:869–77. 10.1530/REP-11-0203 21976616

[8] Berishvili G D’Cotta H Baroiller JF Segner H Reinecke M Differential expression of IGF1 mRNA and peptide in the male and female gonad during early development of a bony fish, the tilapia *Oreochromis niloticus*. Gen Comp Endocrinol (2006) 146:204–10. 10.1016/j.ygcen.2005.11.008 16412440

[9] Hull KL Harvey S. GH as a co-gonadotropin: the relevance of correlative changes in GH secretion and reproductive state. J Endocrinol (2002) 172:1–19. 10.1677/joe.0.1720001 11786370

[10] Rahman MA Mak R Ayad H Smith A Maclean N. Expression of a novel piscine growth hormone gene results in growth enhancement in transgenic tilapia (*Oreochromis niloticus*). Transgenic Res (1998) 7:357–69. 10.1023/A:1008837105299 9859224

[11] Devlin RH Biagi CA Yesaki TY Growth, viability and genetic characteristics of GH transgenic coho salmon strains. Aquaculture (2004) 236:607–32. 10.1016/j.aquaculture.2004.02.026

[12] Nam YK Cho YS Cho HJ Kim DS Accelerated growth performance and stable germ-line transmission in androgenetically derived homozygous transgenic mud loach. Misgurnus mizolepis Aquaculture (2002) 209:257–70. 10.1016/S0044-8486(01)00730-X

[13] Zhong CR Song YL Wang YP Li YM Liao LJ Xie SQ Growth hormone transgene effects on growth performance are inconsistent among offspring derived from different homozygous transgenic common carp (*Cyprinus carpio* L.). Aquaculture (2012) 35(6–357):404–11. 10.1016/j.aquaculture.2012.04.019

[14] Bessey C Devlin RH Liley NR Biagi CA Reproductive performance of growth-enhanced transgenic coho salmon. Trans Am Fish Soc (2004) 133:1205–20. 10.1577/T04-010.1

[15] Fitzpatrick JL Akbarashandiz H Sakhrani D Biagi CA Pitcher TE Devlin RH Cultured growth hormone transgenic salmon are reproductively out-competed by wild-reared salmon in semi-natural mating arenas. Aquaculture (2011) 312:185–91. 10.1016/j.aquaculture.2010.11.044

[16] Rahman MA Ronyai A Engidaw BZ Jauncey K Hwang GL Smith A Growth and nutritional trials on transgenic *Nile tilapia* containing an exogenous fish growth hormone gene. J Fish Biol (2010) 59:62–78. 10.1111/j.1095-8649.2001.tb02338.x

[17] Moreau DTR Conway C Fleming IA. Reproductive performance of alternative male phenotypes of growth hormone transgenic Atlantic salmon (*Salmo salar*). Evol Appl (2011) 4:736–48. 10.1111/j.1752-4571.2011.00196.x 25568019 PMC3352541

[18] Cao M Chen J Peng W Wang Y Liao L Li Y Effects of growth hormone over-expression on reproduction in the common carp *Cyprinus carpio* L. Gen Comp Endocrinol (2014) 195:47–57. 10.1016/j.ygcen.2013.10.011 24184869

[19] Grabherr MG Haas BJ Yassour M Levin JZ Thompson DA Amit I Trinity: reconstructing a full-length transcriptome without a genome from RNA-Seq data. Nat Biotechnol (2011) 29:644–52. 10.1038/nbt.1883 21572440 PMC3571712

[20] Anders S Huber W. Differential expression analysis for sequence count data. Genome Biol (2010) 11:R106. 10.1186/gb-2010-11-10-r106 20979621 PMC3218662

[21] Young MD Wakefield MJ Smyth GK Oshlack A. Gene ontology analysis for RNA-seq: accounting for selection bias. Genome Biol (2010) 11:R14. 10.1186/gb-2010-11-2-r14 20132535 PMC2872874

[22] Xie C Mao X Huang J Ding Y Wu J Dong S KOBAS 2.0: a web server for annotation and identification of enriched pathways and diseases. Nucleic Acids Res (2011) 9:W316–22. 10.1093/nar/gkr483 21715386 PMC3125809

[23] Thisse C Thisse B. High-resolution in situ hybridization to whole-mount zebrafish embryos. Nat Protoc (2008) 3:59–69. 10.1038/nprot.2007.514 18193022

[24] Wu G Chen L Zhong S Li Q Song C Jin B Enzyme-linked immunosorbent assay of changes in serum levels of growth hormone (cGH) in common carps (*Cyprinus carpio*). Sci China C Life Sci (2008) 51:157–63. 10.1007/s11427-008-0022-z 18239894

[25] Ma Q Liu SF Zhuang ZM Sun ZZ Liu CL Su YQ Molecular cloning, expression analysis of insulin-like growth factor I (IGF-I) gene and IGF-I serum concentration in female and male Tongue sole (*Cynoglossus semilaevis*). Comp Biochem Physiol B Biochem Mol Biol (2011) 160:208–14. 10.1016/j.cbpb.2011.08.008 21893211

[26] Maffei M Halaas J Ravussin E Pratley RE Lee GH Zhang Y Leptin levels in human and rodent: measurement of plasma leptin and ob RNA in obese and weight-reduced subjects. Nat Med (1995) 1:1155–61. 10.1038/nm1195-1155 7584987

[27] Peyon P Zanuy S Carrillo M. Action of leptin on in vitro luteinizing hormone release in the European sea bass (*Dicentrarchus labrax*). Biol Reprod (2001) 65:1573–8. 10.1095/biolreprod65.5.1573 11673277

[28] Weil C Le Bail PY Sabin N Le Gac F. *In vitro* action of leptin on FSH and LH production in rainbow trout (*Onchorynchus mykiss*) at different stages of the sexual cycle. Gen Comp Endocrinol (2003) 130:2–12. 10.1016/S0016-6480(02)00504-X 12535619

[29] Pang Y Thomas P Involvement of estradiol-17β and its membrane receptor, G protein coupled receptor 30 (GPR30) in regulation of oocyte maturation in zebrafish, *Danio rerio*. Gen Comp Endocrinol (2009) 161:58–61. 10.1016/j.ygcen.2008.10.003 18952087 PMC2754812

[30] Yu WH Kimura M Walczewska A Karanth S McCann SM Role of leptin in hypothalamic–pituitary function. Proc Natl Acad Sci U S A (1997) 94:1023–8. 10.1073/pnas.94.3.1023 9023376 PMC19633

[31] Trombley S Schmitz M. Leptin in fish: possible role in sexual maturation in male Atlantic salmon. Fish Physiol Biochem (2013) 39:103–6. 10.1007/s10695-012-9731-0 23053613

[32] Zhong C Song Y Wang Y Zhang T Duan M Li Y Increased food intake in growth hormone-transgenic common carp (*Cyprinus carpio* L.) may be mediated by upregulating agouti-related protein (AgRP). Gen Comp Endocrinol (2013) 192:81–8. 10.1016/j.ygcen.2013.03.024 23583469

[33] Riley LG Walker AP Dorough CP Schwandt SE Grau E. Glucose regulates ghrelin, neuropeptide Y, and the GH/IGF-I axis in the tilapia, *Oreochromis mossambicus*. Comp Biochem Physiol A Mol Integr Physiol (2009) 154:541–6. 10.1016/j.cbpa.2009.08.018 19735736

[34] Conde-Sieira M Agulleiro MJ Aguilar AJ Miguez JM Cerda-Reverter JM Soengas JL. Effect of different glycaemic conditions on gene expression of neuropeptides involved in control of food intake in rainbow trout; interaction with stress. J Exp Biol (2010) 213:3858–65. 10.1242/jeb.048439 21037065

[35] Polakof S Mommsen TP Soengas JL. Glucosensing and glucose homeostasis: from fish to mammals. Comp Biochem Physiol B Biochem Mol Biol (2011) 160:123–49. 10.1016/j.cbpb.2011.07.006 21871969

[36] Kling P Jönsson E Nilsen TO Einarsdottir IE Rønnestad I Stefansson SO The role of growth hormone in growth, lipid homeostasis, energy utilization and partitioning in rainbow trout: interactions with leptin, ghrelin and insulin-like growth factor I. Gen Comp Endocrinol (2012) 175:153–62. 10.1016/j.ygcen.2011.10.014 22094208

[37] Devlin RH Johnsson JI Smailus DE Biagi CA Jönsson E Björnsson BT Increased ability to compete for food by growth hormone-transgenic coho salmon *Oncorhynchus kisutch* (Walbaum). Aquaculture Res (1999) 30:479–82. 10.1046/j.1365-2109.1999.00359.x

[38] Guan B Hu W Zhang T Wang Y Zhu Z Metabolism traits of ‘all-fish’ growth hormone transgenic common carp (*Cyprinus carpio* L.). Aquaculture (2008) 284:217–23. 10.1016/j.aquaculture.2008.06.028

[39] Yada T Moriyama S Suzuki Y Azuma T Takahashi A Hirose S Relationships between obesity and metabolic hormones in the “cobalt” variant of rainbow trout. Gen Comp Endocrinol (2002) 128:36–43. 10.1016/S0016-6480(02)00047-3 12270786

[40] Fukamachi S Yada T Mitani H. Medaka receptors for somatolactin and growth hormone: phylogenetic paradox among fish growth hormone receptors. Genetics (2005) 171:1875–83. 10.1534/genetics.105.048819 16143602 PMC1456111

[41] Volkoff H Canosa LF Unniappan S Cerdá-Reverter JM Bernier NJ Kelly SP Neuropeptides and the control of food intake in fish. Gen Comp Endocrinol (2005) 142:3–19. 10.1016/j.ygcen.2004.11.001 15862543

[42] Moschos S Chan JL Mantzoros CS. Leptin and reproduction: a review. Fertil Steril (2002) 77:433–44. 10.1016/S0015-0282(01)03010-2 11872190

[43] Chehab FF Leptin and reproduction: past milestones, present undertakings, and future endeavors. J Endocrinol (2014) 223:T37–48. 10.1530/JOE-14-0413 25118207 PMC4184910

[44] Rønnestad I Nilsen TO Murashita K Angotzi AR Gamst Moen AG Stefansson SO Leptin and leptin receptor genes in Atlantic salmon: cloning, phylogeny, tissue distribution and expression correlated to long-term feeding status. Gen Comp Endocrinol (2010) 168:55–70. 10.1016/j.ygcen.2010.04.010 20403358

[45] Tinoco AB Nisembaum LG Isorna E Delgado MJ de Pedro N. Leptins and leptin receptor expression in the goldfish (*Carassius auratus*). Regulation by food intake and fasting/overfeeding conditions. Peptides (2012) 34:329–35. 10.1016/j.peptides.2012.02.001 22342497

[46] Zhou H Wang X Ko WK Wong AO. Evidence for a novel intrapituitary autocrine/paracrine feedback loop regulating growth hormone synthesis and secretion in grass carp pituitary cells by functional interactions between gonadotrophs and somatotrophs. Endocrinology (2004) 145:5548–59. 10.1210/en.2004-0362 15331572

[47] Levavi-Sivan B Bogerd J Mañanós EL Gómez A Lareyre JJ. Perspectives on fish gonadotropins and their receptors. Gen Comp Endocrinol (2010) 165:412–37. 10.1016/j.ygcen.2009.07.019 19686749

[48] Wong AO Chang JP Peter RE. *In vitro* and *in vivo* evidence that dopamine exerts growth hormone-releasing activity in goldfish. Am J Physiol (1993) 264:925–32. 10.1152/ajpendo.1993.264.6.E925 8101429

[49] Peter RE Chang JP Nahorniak CS Omeljaniuk RJ Sokolowska M Shih SH Interactions of catecholamines and GnRH in regulation of gonadotropin secretion in teleost fish. Recent Prog Horm Res (1986) 42:513–48. 3090658

[50] Dufour S Sebert ME Weltzien FA Rousseau K Pasqualini C. Neuroendocrine control by dopamine of teleost reproduction. J Fish Biol (2010) 76:129–60. 10.1111/j.1095-8649.2009.02499.x 20738703

[51] Popesku JT Mennigen JA Chang JP Trudeau VL. Dopamine D1 receptor blockage potentiates AMPA-stimulated luteinising hormone release in the goldfish. J Neuroendocrinol (2011) 23:302–9. 10.1111/j.1365-2826.2011.02114.x 21276102

[52] Levavi-Sivan B Avitan A Tamir K Characterization of the inhibitory dopamine receptor from the pituitary of tilapia. Fish Physiol Biochem (2013) 28:73–5. 10.1023/B:FISH.0000030479.47055.24

[53] Moussavi M Wlasichuk M Chang JP Habibi HR. Seasonal effect of GnIH on gonadotrope functions in the pituitary of goldfish. Mol Cell Endocrinol (2012) 350:53–60. 10.1016/j.mce.2011.11.020 22155567

[54] Peng W Cao M Chen J Li Y Wang Y Zhu Z GnIH plays a negative role in regulating GtH expression in the common carp, *Cyprinus carpio* L. Gen Comp Endocrinol (2016) 235:18–28. 10.1016/j.ygcen.2016.06.001 27263051

[55] Trudeau VL Peter RE Sloley BD. Testosterone and estradiol potentiate the serum gonadotropin response to gonadotropin-releasing hormone in goldfish. Biol Reprod (1991) 44:951–60. 10.1095/biolreprod44.6.951 1873395

[56] Wang DS Jiao B Hu C Huang X Liu Z Cheng CHK. Discovery of a gonad-specific IGF subtype in teleost. Biochem Biophys Res Commun (2008) 367:336–41. 10.1016/j.bbrc.2007.12.136 18166148

[57] Li J Liu Z Wang D Cheng CHK. Insulin-like growth factor 3 is involved in oocyte maturation in zebrafish. Biol Reprod (2011) 84:476–86. 10.1095/biolreprod.110.086363 21084715

[58] Li J Chu L Sun X Liu Y Cheng CHK. IGFs mediate the action of LH on oocyte maturation in zebrafish. Mol Endocrinol (2015) 29:373–83. 10.1210/me.2014-1218 25584412 PMC4399333

[59] Nóbrega RH Morais RD Crespo D de Waal PP de França LR Schulz RW Fsh stimulates spermatogonial proliferation and differentiation in zebrafish via igf3. Endocrinology (2015) 156:3804–17. 10.1210/en.2015-1157 26207345

