# Supplementary material for: Growth Hormone Overexpression Disrupts Reproductive Status Through Actions on Leptin
Source: Front Endocrinol (Lausanne). 2018 Mar 27;9:131. doi: 10.3389/fendo.2018.00131 (PMC5880896; doi:10.3389/fendo.2018.00131)
Supplement: Supplementary file 1 [file data_sheet_1.docx]

**Supplemental data**


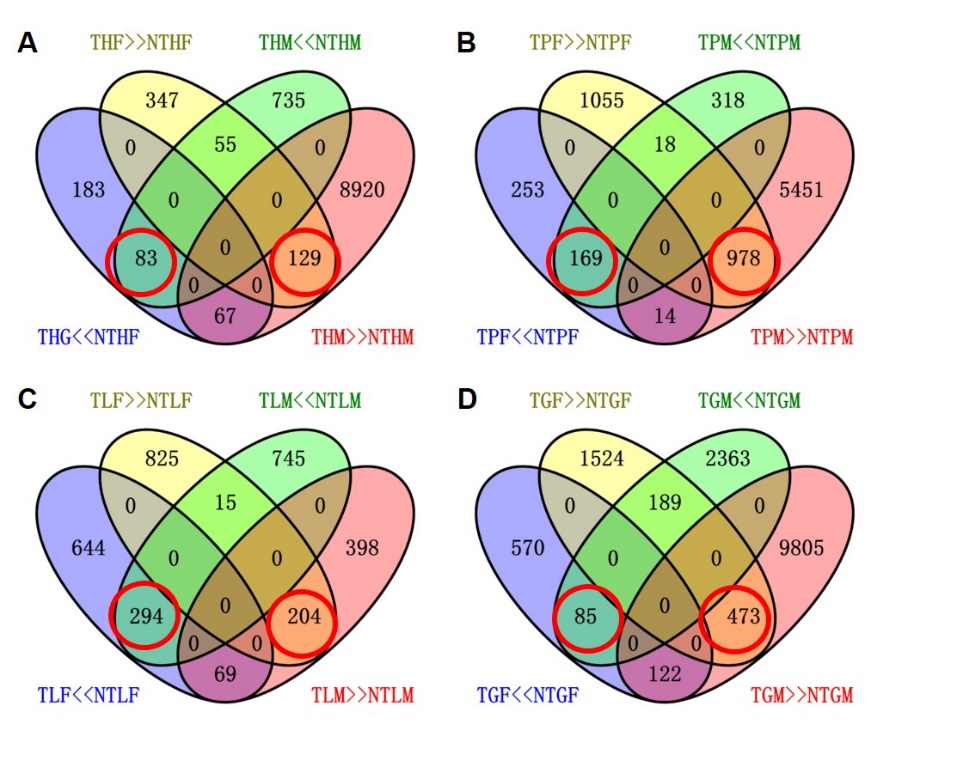


Supplemental Figure 1. Venn diagram of DEGs between different comparisons. Overlapping regions represent DEGs in each comparison. (A) Venn diagram of DEGs between female and male NT and T in the hypothalamus. (B) Venn diagram of DEGs between female and male NT and T in the pituitary. (C) Venn diagram of DEGs between female and male NT and T in the liver. (D) Venn diagram of DEGs between female and male NT and T in the gonad.


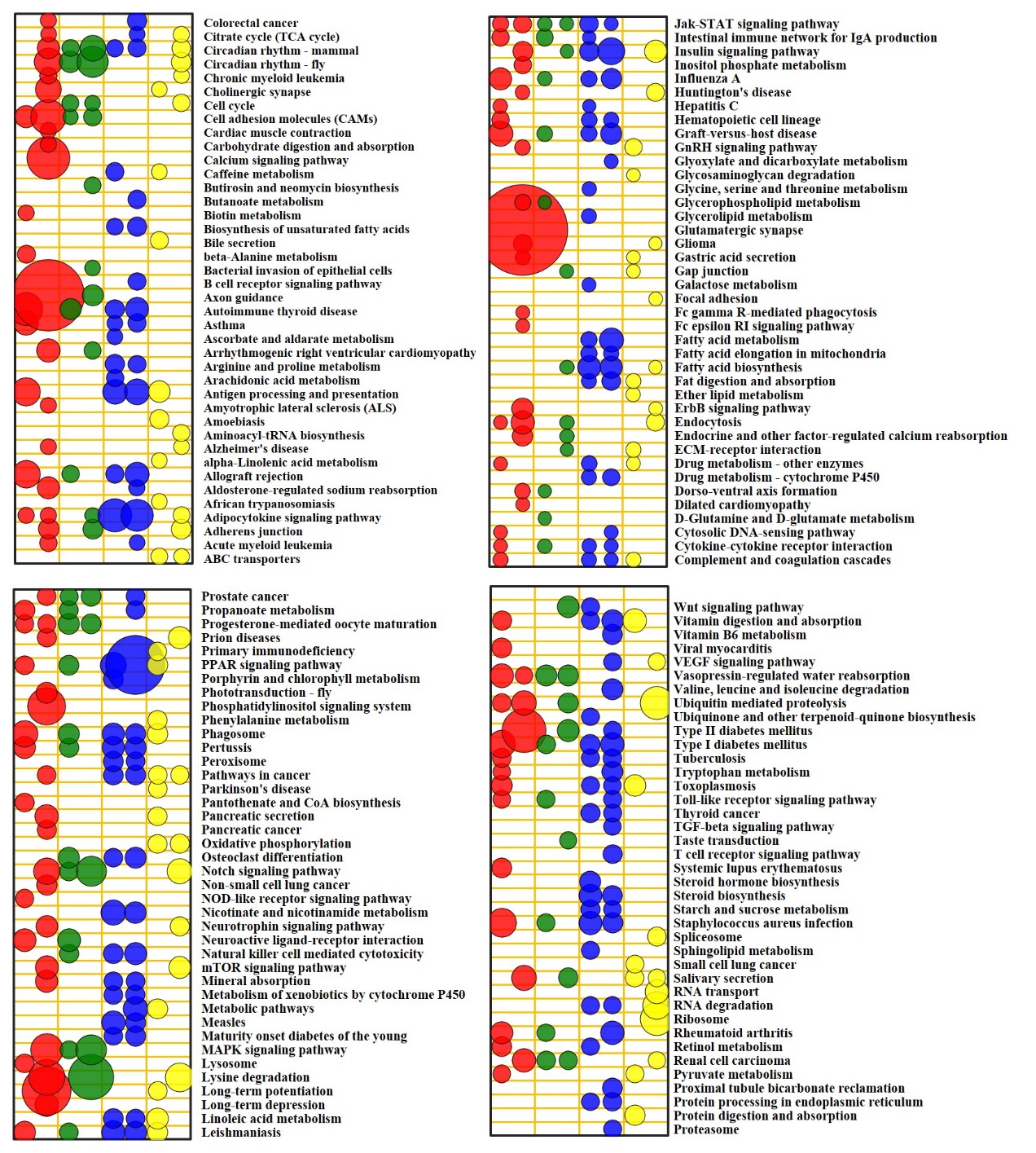
Supplemental Figure 2. Pathways enrichment in different tissues of NT and T common carp in females and male. Red circles represented hypothalamus. Green circles represented pituitary. Blue circles represented liver. Yellow circles represented gonad. The size of the circle represented the *P*-value of each comparison differences between NT and T fish. Bigger circle represented lower *P*-value while smaller circle means higher *P*-value.


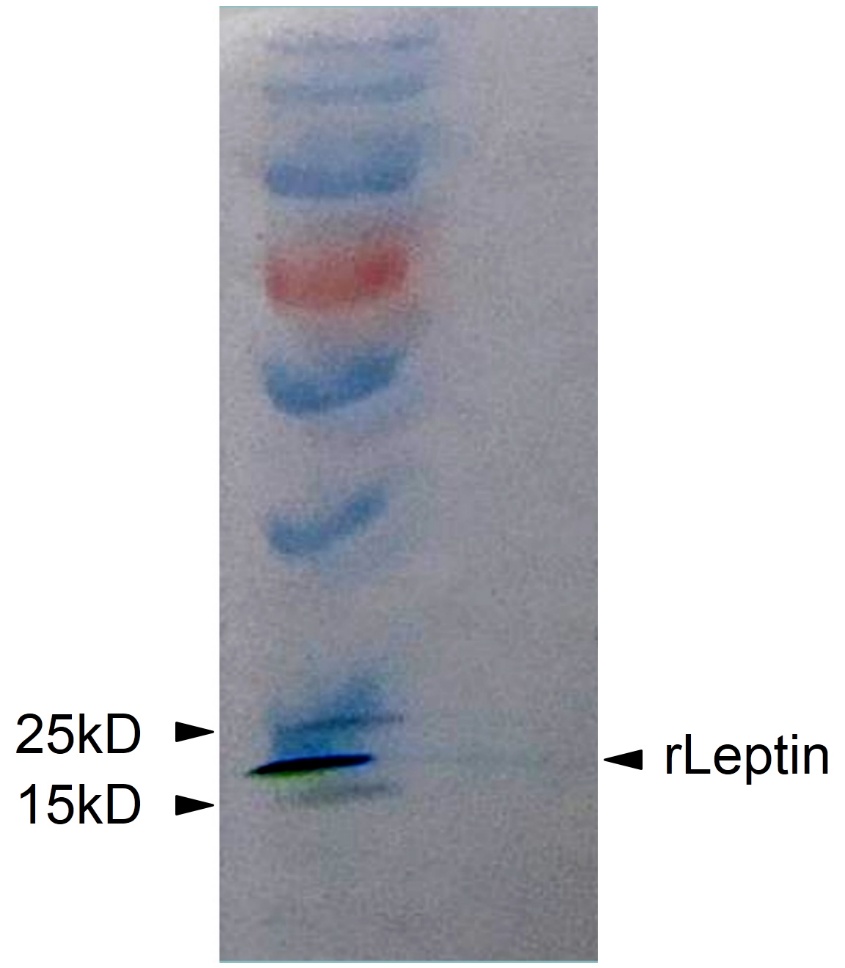


Supplemental Figure 3. Confirmation of the purity of recombinant common carp Leptin protein by polyacrylamide gel electrophoresis.


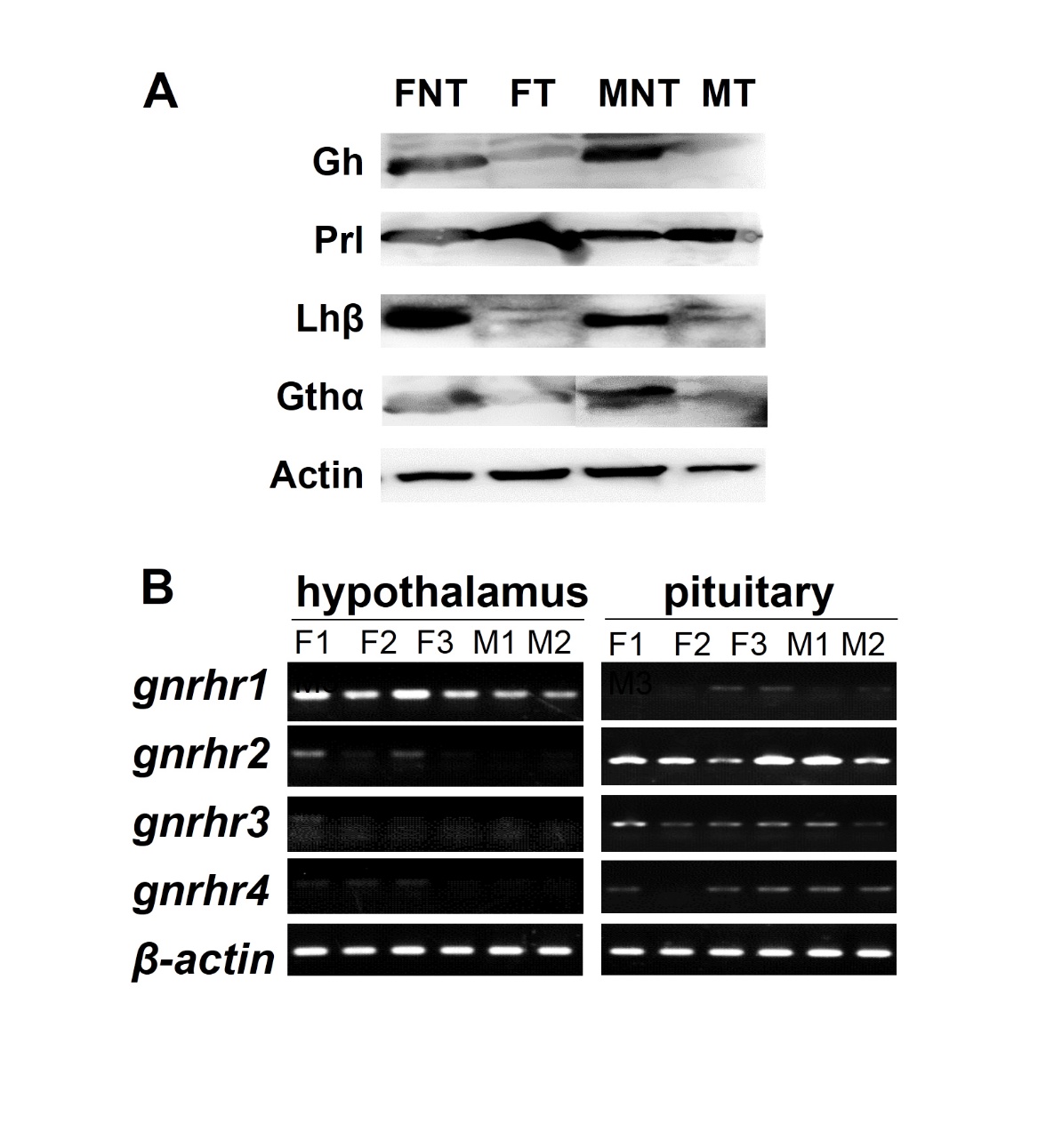


Supplemental Figure 4. Pituitary hormone level and *gnrhr* expression female (F) and male (M) non-transgenic (NT) and GH-transgenic (T) common carp. (A) Levels of pituitary Gh, Prl, Lhβ, Gthα protein in 5-month-old NT and T common carp. (B) Expression the *gnrhr* family in the hypothalamus and pituitary of NT common carp. 3 females and 3 males were randomly selected for replicates.


Supplemental Table 1. Output statistics of sequencing.

| **Samples** | **Total Raw Reads** | **Total Clean Reads** | **Total Clean Nucleotides (nt)** | **Q20 percentage** | **N percentage** | **GC percentage** |
| --- | --- | --- | --- | --- | --- | --- |
| NTGF | 32,364,598 | 27,031,204 | 2,432,808,360 | 97.02% | 0.00% | 51.18% |
| NTHF | 29,135,594 | 26,705,034 | 2,403,453,060 | 98.08% | 0.00% | 47.85% |
| NTHM | 30,028,204 | 27,604,808 | 2,484,432,720 | 98.31% | 0.00% | 45.91% |
| NTLF | 28,208,710 | 26,518,224 | 2,386,640,160 | 98.44% | 0.00% | 48.44% |
| NTLM | 27,464,832 | 25,603,640 | 2,304,327,600 | 97.92% | 0.00% | 48.53% |
| NTPF | 29,808,880 | 27,757,008 | 2,498,130,720 | 98.31% | 0.00% | 46.98% |
| NTPM | 28,009,992 | 25,950,968 | 2,335,587,120 | 98.30% | 0.00% | 46.69% |
| TGF | 32,312,622 | 27,530,338 | 2,477,730,420 | 96.44% | 0.00% | 50.67% |
| THF | 29,471,040 | 26,772,004 | 2,409,480,360 | 98.17% | 0.00% | 47.26% |
| THM | 29,566,028 | 27,044,814 | 2,434,033,260 | 98.14% | 0.00% | 47.65% |
| TLF | 29,804,230 | 27,680,660 | 2,491,259,400 | 98.27% | 0.00% | 48.59% |
| TLM | 27,883,042 | 26,321,480 | 2,368,933,200 | 98.50% | 0.00% | 47.44% |
| TPF | 28,806,194 | 26,527,834 | 2,387,505,060 | 98.19% | 0.00% | 48.10% |
| TPM | 30,111,492 | 27,605,568 | 2,484,501,120 | 98.22% | 0.00% | 48.68% |

Supplemental Table 2. Statistics of assembly quality.

| **assembly quality** | **Sample** | **Total Number** | **Total Length(nt)** | **Mean Length(nt)** | **N50** | **Total Consensus Sequences** | **Distinct Clusters** | **Distinct Singletons** |
| --- | --- | --- | --- | --- | --- | --- | --- | --- |
| Contig | NTGF | 107,710 | 29,026,004 | 269 | 345 | - | - | - |
|  | NTHF | 254,340 | 64,065,231 | 252 | 317 | - | - | - |
|  | NTHM | 244,628 | 60,028,625 | 245 | 302 | - | - | - |
|  | NTLF | 119,696 | 28,761,630 | 240 | 287 | - | - | - |
|  | NTLM | 117,284 | 27,317,544 | 233 | 280 | - | - | - |
|  | NTPF | 228,144 | 55,458,104 | 243 | 298 | - | - | - |
|  | NTPM | 194,298 | 46,269,227 | 238 | 282 | - | - | - |
|  | TGF | 128,133 | 33,729,922 | 263 | 337 | - | - | - |
|  | THF | 262,713 | 66,785,958 | 254 | 320 | - | - | - |
|  | THM | 255,168 | 65,345,390 | 256 | 325 | - | - | - |
|  | TLF | 125,419 | 30,156,058 | 240 | 293 | - | - | - |
|  | TLM | 120,195 | 28,027,750 | 233 | 277 | - | - | - |
|  | TPF | 237,163 | 59,218,624 | 250 | 310 | - | - | - |
|  | TPM | 218,062 | 55,328,330 | 254 | 319 | - | - | - |
| Unigene | NTGF | 56,221 | 26,703,321 | 475 | 645 | 56,221 | 18,639 | 37,582 |
|  | NTHF | 110,996 | 61,495,002 | 554 | 870 | 110,996 | 7,068 | 103,928 |
|  | NTHM | 110,322 | 55,682,901 | 505 | 736 | 110,322 | 6,090 | 104,232 |
|  | NTLF | 55,447 | 26,597,600 | 480 | 671 | 55447 | 2127 | 53320 |
|  | NTLM | 50,057 | 22,305,285 | 446 | 565 | 50,057 | 1,800 | 48,257 |
|  | NTPF | 101,798 | 50,293,923 | 494 | 691 | 101798 | 5314 | 96484 |
|  | NTPM | 91,982 | 41,009,081 | 446 | 572 | 91,982 | 3,746 | 88,236 |
|  | TGF | 63,061 | 30,884,143 | 490 | 678 | 63,061 | 22,233 | 40,828 |
|  | THF | 117,674 | 65,779,780 | 559 | 895 | 117,674 | 7,442 | 110,232 |
|  | THM | 114,605 | 63,651,114 | 555 | 886 | 114,605 | 7,071 | 107,534 |
|  | TLF | 54,479 | 25,956,594 | 476 | 656 | 54479 | 2022 | 52457 |
|  | TLM | 52,348 | 23,511,847 | 449 | 581 | 52,348 | 1,892 | 50,456 |
|  | TPF | 108,091 | 55,186,342 | 511 | 744 | 108091 | 5537 | 102554 |
|  | TPM | 99,781 | 50,896,717 | 510 | 740 | 99,781 | 5,282 | 94,499 |
| All-unigenes | All | 172,823 | 163,196,884 | 944 | 1681 | 172,823 | 142,548 | 30,275 |

Supplemental Table 3. Statistics of mapped rates of reads matched to reference database.

| **Sample** | **Total Reads** | **Total BasePairs** | **Total Mapped Reads** | **perfect match** | **<=2bp mismatch** | **unique match** | **multi-position match** | **Total Unmapped Reads** |
| --- | --- | --- | --- | --- | --- | --- | --- | --- |
| TGF | 25564448 | 2556444800 | 13301600 | 7683170 | 5618430 | 11636240 | 1665360 | 12262848 |
|  | **100.00%** | **100.00%** | **52.03%** | **30.05%** | **21.98%** | **45.52%** | **6.51%** | **47.97%** |
| NTGF | 26301802 | 2630180200 | 13598131 | 7776870 | 5821261 | 11813423 | 1784708 | 12703671 |
|  | **100.00%** | **100.00%** | **51.70%** | **29.57%** | **22.13%** | **44.91%** | **6.79%** | **48.30%** |
| TGM | 26950228 | 2695022800 | 14198460 | 8405855 | 5792605 | 12479843 | 1718617 | 12751768 |
|  | **100.00%** | **100.00%** | **52.68%** | **31.19%** | **21.49%** | **46.31%** | **6.38%** | **47.32%** |
| THF | 26606908 | 2660690800 | 13836320 | 8869325 | 4966995 | 12201270 | 1635050 | 12770588 |
|  | **100.00%** | **100.00%** | **52.00%** | **33.33%** | **18.67%** | **45.86%** | **6.15%** | **48.00%** |
| THM | 26874918 | 2687491800 | 14166960 | 9053249 | 5113711 | 12431260 | 1735700 | 12707958 |
|  | **100.00%** | **100.00%** | **52.71%** | **33.69%** | **19.03%** | **46.26%** | **6.46%** | **47.29%** |
| TLF | 27524928 | 2752492800 | 16514680 | 10515930 | 5998750 | 14372733 | 2141947 | 11010248 |
|  | **100.00%** | **100.00%** | **60.00%** | **38.21%** | **21.79%** | **52.22%** | **7.78%** | **40.00%** |
| TML | 26186124 | 2618612400 | 15064042 | 9638326 | 5425716 | 13114452 | 1949590 | 11122082 |
|  | **100.00%** | **100.00%** | **57.53%** | **36.81%** | **20.72%** | **50.08%** | **7.45%** | **42.47%** |
| TPF | 26390180 | 2639018000 | 13929425 | 8992976 | 4936449 | 12404130 | 1525295 | 12460755 |
|  | **100.00%** | **100.00%** | **52.78%** | **34.08%** | **18.71%** | **47.00%** | **5.78%** | **47.22%** |
| TPM | 27465070 | 2746507000 | 14813673 | 9459769 | 5353904 | 13250741 | 1562932 | 12651397 |
|  | **100.00%** | **100.00%** | **53.94%** | **34.44%** | **19.49%** | **48.25%** | **5.69%** | **46.06%** |
| NTGM | 25497306 | 2549730600 | 12573362 | 7446048 | 5127314 | 11280045 | 1293317 | 12923944 |
|  | **100.00%** | **100.00%** | **49.31%** | **29.20%** | **20.11%** | **44.24%** | **5.07%** | **50.69%** |
| NTHF | 26537702 | 2653770200 | 14292745 | 9252655 | 5040090 | 12566822 | 1725923 | 12244957 |
|  | **100.00%** | **100.00%** | **53.86%** | **34.87%** | **18.99%** | **47.35%** | **6.50%** | **46.14%** |
| NTHM | 27458560 | 2745856000 | 14597694 | 9848803 | 4748891 | 13094096 | 1503598 | 12860866 |
|  | **100.00%** | **100.00%** | **53.16%** | **35.87%** | **17.29%** | **47.69%** | **5.48%** | **46.84%** |
| NTLF | 26359068 | 2635906800 | 14988897 | 9391417 | 5597480 | 13267188 | 1721709 | 11370171 |
|  | **100.00%** | **100.00%** | **56.86%** | **35.63%** | **21.24%** | **50.33%** | **6.53%** | **43.14%** |
| NTML | 25454592 | 2545459200 | 14955527 | 9274972 | 5680555 | 13197165 | 1758362 | 10499065 |
|  | **100.00%** | **100.00%** | **58.75%** | **36.44%** | **22.32%** | **51.85%** | **6.91%** | **41.25%** |
| NTPF | 27633822 | 2763382200 | 14407862 | 9273462 | 5134400 | 12803547 | 1604315 | 13225960 |
|  | **100.00%** | **100.00%** | **52.14%** | **33.56%** | **18.58%** | **46.33%** | **5.81%** | **47.86%** |
| NTPM | 25840578 | 2584057800 | 13433638 | 8724357 | 4709281 | 12051218 | 1382420 | 12406940 |
|  | **100.00%** | **100.00%** | **51.99%** | **33.76%** | **18.22%** | **46.64%** | **5.35%** | **48.01%** |
| **average** | **100.00%** | **100.00%** | **53.83%** | **33.79%** | **20.05%** | **47.55%** | **6.29%** | **46.16%** |
|  |  |  |  |  |  |  |  |  |
| **Sample** | **Total Reads** | **Total BasePairs** | **Total Mapped Reads** | **perfect match** | **<=2bp mismatch** | **unique match** | **multi-position match** | **Total Unmapped Reads** |
| TGF | 25564448 | 2556444800 | 10152009 | 5685719 | 4466290 | 8637284 | 1514725 | 15412439 |
|  | **100.00%** | **100.00%** | **39.71%** | **22.24%** | **17.47%** | **33.79%** | **5.93%** | **60.29%** |
| NTGF | 26301802 | 2630180200 | 10561679 | 6041050 | 4520629 | 8903088 | 1658591 | 15740123 |
|  | **100.00%** | **100.00%** | **40.16%** | **22.97%** | **17.19%** | **33.85%** | **6.31%** | **59.84%** |
| TGM | 26950228 | 2695022800 | 10676607 | 6120540 | 4556067 | 9247104 | 1429503 | 16273621 |
|  | **100.00%** | **100.00%** | **39.62%** | **22.71%** | **16.91%** | **34.31%** | **5.30%** | **60.38%** |
| THF | 26606908 | 2660690800 | 9164449 | 5599471 | 3564978 | 7914427 | 1250022 | 17442459 |
|  | **100.00%** | **100.00%** | **34.44%** | **21.05%** | **13.40%** | **29.75%** | **4.70%** | **65.56%** |
| THM | 26874918 | 2687491800 | 9574566 | 5823497 | 3751069 | 8257898 | 1316668 | 17300352 |
|  | **100.00%** | **100.00%** | **35.63%** | **21.67%** | **13.96%** | **30.73%** | **4.90%** | **64.37%** |
| TLF | 27524928 | 2752492800 | 12432185 | 7769875 | 4662310 | 10463153 | 1969032 | 15092743 |
|  | **100.00%** | **100.00%** | **45.17%** | **28.23%** | **16.94%** | **38.01%** | **7.15%** | **54.83%** |
| TML | 26186124 | 2618612400 | 10903238 | 6904255 | 3998983 | 9201770 | 1701468 | 15282886 |
|  | **100.00%** | **100.00%** | **41.64%** | **26.37%** | **15.27%** | **35.14%** | **6.50%** | **58.36%** |
| TPF | 26390180 | 2639018000 | 10032432 | 6059411 | 3973021 | 8673639 | 1358793 | 16357748 |
|  | **100.00%** | **100.00%** | **38.02%** | **22.96%** | **15.05%** | **32.87%** | **5.15%** | **61.98%** |
| TPM | 27465070 | 2746507000 | 10935781 | 6491495 | 4444286 | 9514398 | 1421383 | 16529289 |
|  | **100.00%** | **100.00%** | **39.82%** | **23.64%** | **16.18%** | **34.64%** | **5.18%** | **60.18%** |
| NTGM | 25497306 | 2549730600 | 7981482 | 4522692 | 3458790 | 6959821 | 1021661 | 17515824 |
|  | **100.00%** | **100.00%** | **31.30%** | **17.74%** | **13.57%** | **27.30%** | **4.01%** | **68.70%** |
| NTHF | 26537702 | 2653770200 | 9436375 | 5760535 | 3675840 | 8114673 | 1321702 | 17101327 |
|  | **100.00%** | **100.00%** | **35.56%** | **21.71%** | **13.85%** | **30.58%** | **4.98%** | **64.44%** |
| NTHM | 27458560 | 2745856000 | 8094599 | 5074969 | 3019630 | 7058433 | 1036166 | 19363961 |
|  | **100.00%** | **100.00%** | **29.48%** | **18.48%** | **11.00%** | **25.71%** | **3.77%** | **70.52%** |
| NTLF | 26359068 | 2635906800 | 11206272 | 6910986 | 4295286 | 9737775 | 1468497 | 15152796 |
|  | **100.00%** | **100.00%** | **42.51%** | **26.22%** | **16.30%** | **36.94%** | **5.57%** | **57.49%** |
| NTML | 25454592 | 2545459200 | 11325344 | 6978110 | 4347234 | 9771868 | 1553476 | 14129248 |
|  | **100.00%** | **100.00%** | **44.49%** | **27.41%** | **17.08%** | **38.39%** | **6.10%** | **55.51%** |
| NTPF | 27633822 | 2763382200 | 10222970 | 6218616 | 4004354 | 8715060 | 1507910 | 17410852 |
|  | **100.00%** | **100.00%** | **36.99%** | **22.50%** | **14.49%** | **31.54%** | **5.46%** | **63.01%** |
| NTPM | 25840578 | 2584057800 | 9025609 | 5437340 | 3588269 | 7712944 | 1312665 | 16814969 |
|  | **100.00%** | **100.00%** | **34.93%** | **21.04%** | **13.89%** | **29.85%** | **5.08%** | **65.07%** |
| **average** | **100.00%** | **100.00%** | **38.09%** | **22.93%** | **15.16%** | **32.71%** | **5.38%** | **61.91%** |

Supplemental Table 4. Summary of DEGs in different comparisons.

| group | up-regulated | down-regulated | Total |
| --- | --- | --- | --- |
| NTHF vs THF | 531 | 333 | 864 |
| NTHM vs THM | 9116 | 873 | 9989 |
| NTPF vs TPF | 2051 | 436 | 2487 |
| NTPM vs TPM | 6443 | 505 | 6948 |
| NTLF vs TLF | 1044 | 1007 | 2051 |
| NTLM vs TLM | 671 | 1054 | 1725 |
| NTGF vs TGF | 2186 | 777 | 2963 |
| NTGM vs TGM | 10400 | 2637 | 13037 |
| NTHF vs THF & NTHM vs THM | 128 | 83 | 211 |
| NTPF vs TPF & NTPM vs TPM | 978 | 168 | 1146 |
| NTLF vs TLF & NTLM vs TLM | 204 | 294 | 498 |
| NTGF vs TGF & NTGM vs TGM | 473 | 85 | 558 |

Supplemental Table 5. DEGs in both male and female of different tissues.

Supplemental Table 6. GO cluster analysis of DEGs in both male and female of different tissues.

Supplemental Table 7. KEGG analysis of DEGs enriched in both male and female of different tissues.

Supplemental Table 8. Filtered DEGs list in different tissues.

Supplemental Table 9. Primers used in the PCR analysis. The PCR primer sequences used for quantification of the genes are listed. The primers were designed spanning the exon-exon borders and amplify PCR products between 100- 250 base pairs (bp) long. Amplified PCR products of all actual cDNAs were sequenced to ensure that the correct mRNA sequences were quantified.

| primer name | | sequence |
| --- | --- | --- |
| gnrhr1-R | GCATCCTCCTGTTCCTACGG | |
| gnrhr1-F | GCCATGTGCAAGATCCTCTG | |
| gnrhr2-R | AGGAAACAGTGTCCCAGTCC | |
| gnrhr2-F | GTGTTCTCCAGCTCAAAGGC | |
| cyp19a1a-F | CATCATACTGAATGTGGGTC | |
| cyp19a1a-R | CGAACGGCTGAAAGAA | |
| cyp19a1b-F | ATAGGCGAGCGGGATGTAGAGT | |
| cyp19a1b-R | AAGTAACGACTGGGAACGGTGT | |
| drd1-F | CCATTTCCAGCCCGTTCC | |
| drd1-R | CGCAGTTGTCCGTCAGCA | |
| drd2-F | CGAACTGCTCGGTGTCTC | |
| drd2-R | CAGGTAATTGGTGGTGGTCT | |
| drd3-F | CGTCAAGGCAGTGGTAAA | |
| drd3-R | AGGAGGCAGTAGCGAGTT | |
| drd4-F | GTAGAAGCCTGTCTTTGCG | |
| drd4-R | CTCTGCGTCCGATACACT | |
| fsh-F | CTTCGTTGTTATGGTGAT | |
| fsh-R | AGTGTTTGGTTTCCTTGT | |
| lh-F | CGACTGTACGATTGAAAG | |
| lh-R | AAGTGGCACAGTGTTATT | |
| gtha-F | ATCAGTGTATGGGATGCT | |
| gtha-R | GATGACAAAATAAATGGG | |
| gnih-F | CAGATTGCCACTTCCTGATGACA | |
| gnih-R | CTGCTGGTTGGGGCTACATTGAG | |
| β-actin-F | GATGATGAAATTGCCGCACTG | |
| β-actin-R | ACCAACCATGACACCCTGATGT | |
| igf3-F | CTCGTGGGAAAGGGATC | |
| igf3-R | TCTGGTATTGCCTCAGAAAC | |
| leptinI-F | GCCAAACCATCTGTCCTTG | |
| leptinI-R | GTCTGACCTCGCTGCTTCT | |
| pkm2-F | CAGTGGTATCAACGCAGAG | |
| pkm2-R | GAATAAAGCGAAATCACATC | |
| mch-F | CCAATCTGCTGAGGACCC | |
| mch-R | CCCTTCTGATGATGGCG | |
| gnrh3-F | GTCATACGGTTGGCTTCC | |
| gnrh3-R | GCGTCCACCTCATTCACT | |
| gh-F | TGCTATGTCGGTGGT | |
| gh-R | CTGTCTGCGTTCCTCA | |

Supplemental Table 10. Validation of DEGs by qPCR. Fifteen DEGs were selected for qPCR analysis and compared with the equivalent RNA-seq data. The data from qPCR were presented as log_2_ fold change.
